# Explicit temporal inductive biases for video moment retrieval via multi-scale Gaussian priors

**DOI:** 10.1371/journal.pone.0354458

**Published:** 2026-09-01

**Authors:** Guanlin Dai, Da Gao, Chao Zheng

**Affiliations:** China Telecom Research Institute, Beijing, China; Xidian University, CHINA

## Abstract

Video moment retrieval aims to localize video segments corresponding to natural language queries. While Transformer models are widely applied, their localization performance declines when processing diverse temporal scales. This limitation arises from the dependence on implicitly learned decoder queries. Without explicit temporal grounding, these unstructured embeddings lead the decoder to perform a global search across the full video sequence. We propose Gaussian-DETR to address this by introducing explicit temporal awareness into the decoding process. Our framework replaces unstructured embeddings with queries constructed from multi-scale Gaussian temporal priors, parameterized by a learnable center and scale. To adapt these priors to the input, we design a multi-scale Gaussian pooling mechanism that extracts local features from the encoder for query initialization, introducing a temporal inductive bias while maintaining the architectural efficiency of DETR. On the QVHighlights benchmark, Gaussian-DETR provides a scale-specific benefit for long-event retrieval, improving Long mAP by +1.98 points over Moment-DETR with statistical support (*p* = 0.0111), while the differences in overall recall are statistically inconclusive. Ablations suggest that the Gaussian prior improves scale-aware query initialization by balancing long-event coverage and local temporal specificity. This mechanism provides a parameter-efficient component for studying scale-aware query initialization in DETR-like video moment retrieval models.

## Introduction

Video Moment Retrieval (VMR) aims to accurately localize the temporal boundaries of specific events in untrimmed video streams via natural language queries [1,2]. Recent large vision-language models and related methods [3–6] have broadened multimodal video understanding. In this work, we focus on efficient DETR-based sequence prediction models for VMR [7–11]. These models combine efficient inference with set-based prediction, enabling cross-modal feature interaction and temporal boundary prediction without heuristic post-processing such as Non-Maximum Suppression (NMS) [12].

However, this end-to-end architecture still faces limitations when processing events with extreme temporal spans. Existing DETR-like models often exhibit localization deviations and metric variability when dealing with extremely long-duration events or transient, short actions. Fundamentally, mainstream models mostly rely on implicitly learned decoder queries. During initialization, these queries do not contain any physical concepts regarding “temporal center” or “temporal span”, causing the model to conduct an unfocused search over the entire video sequence during the decoding phase. This lack of explicit temporal center-and-span parameterization can reduce localization accuracy for temporally localized evidence.

In the image object detection domain, DAB-DETR [13] demonstrated that injecting explicit anchor coordinates into decoder queries substantially improves localization accuracy and training stability. Inspired by this finding, our work extends the principle of structured query parameterization to the one-dimensional temporal axis of video moment retrieval. However, because video temporal content exhibits strong context dependency that differs from static spatial arrangements, a direct adaptation of two-dimensional spatial anchors is insufficient. We therefore design multi-scale Gaussian temporal priors that jointly encode temporal center and span information.

Previous work has also explored Gaussian functions for temporal modeling, but mainly as proposal-generation or sample-mining mechanisms under weak supervision. For example, Zheng et al. [14] use learnable Gaussian functions to generate temporal proposals for contrastive learning, and D3G [15] introduces dynamic Gaussian priors to assist weakly supervised temporal grounding. In contrast to these methods, which use Gaussian distributions as auxiliary tools for proposal scoring or loss computation, our method integrates multi-scale Gaussian priors directly into the decoder query initialization of a fully supervised DETR-style framework.

To this end, we propose Gaussian-DETR as a parameter-efficient mechanism for scale-aware query initialization in DETR-like architectures. Discarding traditional unstructured embeddings, we instantiate each query as a multi-scale Gaussian prior with clear physical meaning by introducing learnable “temporal center” and “scale” parameters. To enable these temporal hypotheses to dynamically adapt to the current input video instances, we further design a multi-scale Gaussian pooling mechanism. This mechanism extracts context-aware local representations from the encoded features and fuses them with semantic prototypes, thereby achieving structured, dynamic query initialization. This design preserves the end-to-end, post-processing-free inference style of DETR while changing the decoder initialization from an unconstrained implicit search to a scale-aware structured hypothesis.

To evaluate this mechanism, we designed it as a lightweight plug-in component and integrated it into Moment-DETR and QD-DETR-style architectures. This cross-model experimental setup provides an additional compatibility check beyond a single baseline implementation, helping to isolate the contribution of the explicit temporal prior. Experiments show that the main benefit is scale-specific: the method provides statistically supported improvement on long-event retrieval, while its effect on overall recall is not statistically conclusive.

The main contributions of this study are summarized as follows:

- Proposed the Gaussian-DETR framework: Inspired by the structured query parameterization of DAB-DETR, we adapt the principle of explicit coordinate-based priors from the spatial to the temporal domain. By introducing multi-scale Gaussian temporal priors into decoder queries, we provide the model with a physically meaningful temporal inductive bias;
- Provided statistical evaluation for long-event retrieval: Across 15 matched seeds, Gaussian-DETR yields a statistically supported improvement in Long mAP under the R1@0.5-selected checkpoint protocol;
- Demonstrated architectural compatibility: Experiments suggest that the multi-scale Gaussian prior module can be integrated into an additional DETR-like architecture, represented by QD-DETR.

## Related work

### Video moment retrieval

The core task of video moment retrieval is to accurately localize the corresponding semantic segments in a video based on natural language descriptions, as studied in early work such as TALL and MCN [1,2]. Proposal-based approaches commonly construct candidate temporal clips, for example with sliding windows, and then match, rank, or refine these candidates using cross-modal scoring and boundary regression modules [1]. Such proposal-centric pipelines rely on heuristic candidate construction or post-processing steps and struggle to balance computational efficiency and localization accuracy when processing long videos.

With the widespread adoption of Transformers, the end-to-end paradigm has gradually become mainstream. Methods represented by Moment-DETR [7] borrow the DETR architecture from object detection, utilizing a set of learnable queries to directly perform cross-attention interactions with video-text features, significantly simplifying the localization pipeline. However, this architecture still has limitations: queries in the model are usually modeled as implicit semantic vectors, lacking explicit temporal position or span priors. This makes the model prone to structural shortcomings when handling long videos or complex events with significant disparities in temporal span.

Recent studies attempt to alleviate this issue from the perspective of decoding mechanisms, training strategies, or query-aware encoding [16]. For example, Sim-DETR reduces query conflicts during decoding by restricting mutual interference among queries and introducing alignment constraints [17]; length-aware methods such as Length-Aware DETR with MomentMix and Length Matters [11,18] utilize length-conditioned decoding or data augmentation to improve localization for short segments. In parallel, QD-DETR [9] improves encoding quality by constructing query-dependent video representations through query-video interaction. These improvements mainly focus on late-stage decoding computations, training regularization, or encoder-side query conditioning, while query initialization remains largely based on learned embeddings without explicit temporal structure. Gaussian-DETR instead focuses on the structural hypotheses used to initialize decoder queries under extreme scale disparities.

### Temporal modeling and proposal generation based on Gaussian distributions

To enhance the model’s ability to model temporal structural continuity and span variations, previous research has attempted to introduce Gaussian distributions or Gaussian mixture models [19] as explicit temporal priors. Such methods are particularly common in weakly supervised video moment retrieval tasks: some works utilize learnable Gaussian functions to characterize the temporal center and duration range of events, generate high-quality proposal segments, and assist in the mining and alignment of positive and negative samples [14]. Studies like D3G [15] combine minimal “glance-level” annotations and introduce dynamic Gaussian priors to improve the model’s robustness and stability in weakly supervised scenarios.

The aforementioned methods demonstrate the value of Gaussian functions for temporal structure modeling, primarily as auxiliary mechanisms for proposal generation, sample selection, or loss constraints. Gaussian-DETR uses this parameterization differently: it models Gaussian priors as part of the decoder query hypothesis space in a fully supervised DETR-style framework. This shifts the role of Gaussian temporal modeling from indirect proposal or scoring support to explicit query construction. Directly encoding temporal center and span information into decoder queries provides a structured way to study scale disparities between long and short events.

### Multi-scale temporal modeling and structural priors

Handling events of varying lengths in videos is a core challenge in temporal localization. To capture temporal spans of different scales, previous works mostly focused on the feature extraction stage, such as utilizing temporal pyramids, ActionFormer’s multiscale representation with local self-attention [20], 2D-TAN’s start-end temporal map for adjacent moment relations [21], or multi-resolution feature fusion.

Existing multi-scale modeling is mostly limited to the feature encoding level. Entering the decoding phase, models still rely on randomly initialized or globally shared query variables. This implies that the decoder must perform unguided attention computations over the global video sequence, lacking a clear orientation toward specific temporal centers and span ranges. When confronting lengthy videos or extremely unevenly distributed events, this decoding process—devoid of explicit temporal inductive biases—not only converges slowly but also easily falls into local optima, limiting the final localization precision.

### Transformer query initialization strategies

In Transformer-based architectures, the initialization strategy of queries has a decisive impact on model convergence and final performance. In the field of image object detection, injecting explicit spatial position priors, such as anchor boxes or reference points in DAB-DETR [13], into queries has been widely proven to substantially improve localization accuracy and training stability.

However, this initialization strategy based on explicit positional priors has not been fully extended to the video moment retrieval domain. Current mainstream methods still tend to initialize queries using random vectors or globally learnable parameters that lack physical meaning. Since images are static two-dimensional spaces, while videos possess complex temporal dynamics and context dependencies, the “two-dimensional spatial priors” from the image domain cannot be simply mechanically applied to the “one-dimensional temporal axis” of videos. Temporal localization models require an initialization approach specifically tailored for the temporal dimension—that is, encoding information with clear physical meanings like “temporal center point” and “span length” into the queries, and combining them with the specific features of the current input video, thereby providing the decoder with a clearer, more accurate joint initialization starting point.

### Large vision-language models for temporal grounding

Recent advances in large vision-language models (VLMs) have introduced new approaches to temporal grounding and long-video understanding. Time-R1 [4] improves temporal video grounding through reinforcement-learning post-training with verifiable rewards, whereas LVAgent [6] uses multi-round collaboration among MLLM agents for long-video understanding. While these approaches demonstrate strong zero-shot or few-shot generalization, they typically require substantially more computational resources and depend on large-scale pre-training or multi-agent inference. Our work operates in a complementary regime: we propose a lightweight structural prior that can be integrated into efficient DETR-style architectures using pre-extracted features. The Gaussian temporal prior addresses the specific challenge of multi-scale query initialization within a fixed-parameter model, rather than relying on the emergent reasoning capacity of foundation models.

### Positioning of the proposed method

This paper proposes a Transformer query initialization method based on multi-scale Gaussian priors and constructs the Gaussian-DETR framework for fully supervised video moment retrieval. Our work builds on two lines of prior research. From the structured query initialization paradigm exemplified by DAB-DETR [13], we adopt the principle that explicit coordinate-based priors improve query quality; we adapt this principle from two-dimensional spatial anchors to one-dimensional temporal centers and spans. From the temporal Gaussian modeling literature [14,15], we draw on the idea that Gaussian functions provide a natural parameterization for temporal extent; we repurpose this parameterization from auxiliary proposal or loss mechanisms to the core decoder query construction.

Distinct from spatial priors in the image domain, and considering the strong context dependency of video temporal content, this paper combines instance-level contextual features extracted via Gaussian pooling with learnable semantic prototype embeddings to achieve a synergistic initialization of position and content priors. Before entering the decoding phase, queries already possess temporal structural constraints and instance-specific semantic conditions, reducing the need for unconstrained global searches by the decoder along the temporal axis.

Unlike previous practices that treat Gaussian distributions as proposal generation or training auxiliary tools, Gaussian-DETR incorporates them as structural priors in the decoder query initialization. This design operates solely at the query construction stage and does not modify the encoder, decoder, loss functions, or matching rules of the underlying DETR architecture, supporting integration into the evaluated DETR-style architectures.

## Method

**Fig 1:**
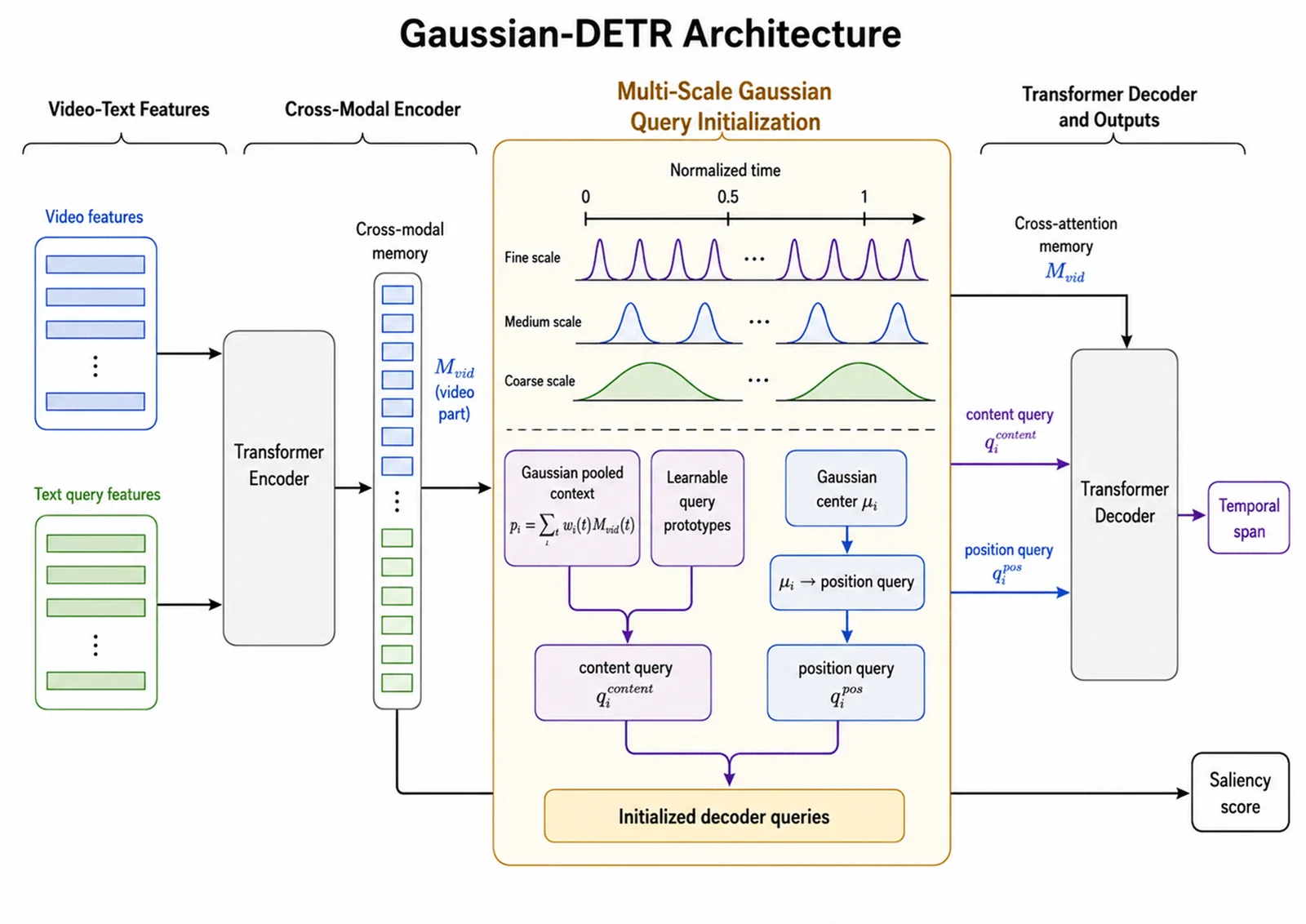
Architecture diagram of the proposed Gaussian-DETR framework. The framework leverages multi-scale Gaussian pyramid pooling and dynamic context-aware query initialization to equip decoder queries with explicit temporal prior coordinates (center and scale) alongside instance-specific contextual representation, avoiding unguided global decoding search.

This paper proposes the Gaussian-DETR framework, which enhances the video segment retrieval capability of the model by introducing explicit temporal priors into the Transformer decoder. Standard DETR-like methods mostly rely on learnable query embeddings that lack explicit physical meaning. In contrast, our method constructs queries based on a multi-scale Gaussian pyramid, enabling the model to more effectively capture video events with drastically varying temporal spans. The overall architecture of our proposed framework is illustrated in Fig 1.

### Multi-scale Gaussian pyramid pooling

Original video streams contain numerous redundant frames, whereas the task objective is solely to extract key action segments. This study designs a multi-scale Gaussian pyramid pooling module as a temporal bottleneck layer to compress and map a video feature sequence *V* of arbitrary length into a fixed number (*N* = 112) of query vectors. This module regularizes the input space of the Transformer decoder and introduces multi-scale inductive biases to the model.

The module adopts a set of hierarchical, learnable Gaussian kernels as a soft attention mechanism, replacing traditional uniform sampling. In the implementation, the *T* temporal positions of the input feature tensor are placed on a uniformly spaced closed interval, $\tau_{t}=(t-1)/(T-1)$ for t=1,...,T, matching the temporal grid generated by torch.linspace(0, 1, T). For the *i*-th kernel at pyramid level *l*, its response weight is computed over $\tau_{t}$ as:


$$\begin{array}{c} {\tilde{w}}_{l,i}(t)=\exp\left(-\frac{(\tau_{t}-\mu_{l,i}{)}^{2}}{2\sigma_{l,i}^{2}}\right)\cdot m(t),\;w_{l,i}(t)=\frac{{\tilde{w}}_{l,i}(t)}{\sum_{t^{\prime }=1}^{T}{\tilde{w}}_{l,i}(t^{\prime })+\epsilon } \end{array}$$ (1)

where *m*(*t*) is a binary mask that excludes padded frames, and $\epsilon =10^{-6}$ ensures numerical stability. Each kernel is parameterized by a learnable normalized temporal center $\mu_{l,i}\in [0,1]$ (constrained via a sigmoid mapping from an unconstrained parameter) and a learnable temporal span $\sigma_{l,i}$ in the same normalized coordinate system. In implementation, the raw span parameter $\rho_{l,i}$ is unconstrained and mapped to a positive span as $\sigma_{l,i}=|\rho_{l,i}|+10^{-6}$ for numerical stability. Through the discrete weighted aggregation $q_{l,i}=\sum_{t=1}^{T}w_{l,i}(t)\cdot V_{t}$, a series of context-aware feature descriptors is obtained.

To capture temporal structure at multiple granularities, we construct a three-level pyramid. Each level initializes $\sigma_{l,i}$ proportionally to the reciprocal of its kernel count, scaled by a level-specific factor $s_{l}$, i.e., $\sigma_{l,i}=s_{l}/N_{l}$. The configuration is summarized in Table 1.

**Table 1:** Multi-scale Gaussian pyramid configuration.

Level	$N_{l}$	Scale factor $s_{l}$	Initial $\sigma_{l,i}$
Fine-grained	64	1.0	≈0.016
Medium-grained	32	1.0	≈0.031
Coarse-grained	16	2.0	≈0.125

The fine-grained level uses narrow kernels to provide dense local temporal hypotheses for short-duration segments, while the coarse-grained level uses wider kernels to aggregate long-range context. Both $\mu_{l,i}$ and $\sigma_{l,i}$ are optimized end-to-end during training.

#### Design rationale for the multi-scale pyramid configuration.

The choice of three pyramid levels with counts *N*_1_ = 64, *N*_2_ = 32, *N*_3_ = 16 (total 112 queries) is guided by three considerations.

**Hierarchical temporal coverage:** The pyramid decomposes temporal modeling into three complementary scales corresponding to different event durations. Fine-grained queries use narrow kernels for local temporal neighborhoods, medium-grained queries cover intermediate ranges, and coarse-grained queries aggregate broader temporal context. This design mirrors multi-scale feature hierarchies in convolutional networks, enabling the decoder to simultaneously attend to temporal details at multiple granularities.

**Query budget trade-off:** Increasing the total query count incurs non-trivial computational overhead and convergence challenges. As shown in the computational complexity analysis, scaling from 10 queries (Moment-DETR baseline) to 112 queries (Gaussian-DETR) increases model-level MAC operations by 62%, mainly because decoder computation scales with the number of queries and the Gaussian initialization adds an additional pooling step. The 64/32/16 split therefore represents a practical balance: dense fine-grained sampling for short-duration events, progressively sparser sampling for coarse contextual coverage, within a query budget that remains tractable for training and inference.

**Controlled ablation design:** To isolate the contribution of the fine-grained scale from the confounding effect of query quantity, we conduct a fixed-total-query ablation study in the ablation section that removes the fine-grained level (*N*_1_ = 0) while redistributing queries to the medium (*N*_2_ = 80) and coarse (*N*_3_ = 32) levels, maintaining the total at 112. This controlled experiment evaluates whether hierarchical scale structure provides value beyond simply having more queries.

### Dynamic context-aware query initialization

Standard DETR architectures typically rely on static query embeddings that are agnostic to the input video. However, efficient temporal localization requires query vectors to be conditioned on specific video instances during the initialization stage. Combining the aforementioned Gaussian pyramid pooling, this paper designs a context-aware dynamic query generation mechanism to simultaneously inject semantic content and positional priors into the Transformer decoder.

The final query input to the decoder $Q\in {\mathbb{R}}^{N_{\text{total}}\times D}$ is formulated as a fusion of two complementary information sources: learnable semantic prototypes and video-specific contexts. Let $C\in {\mathbb{R}}^{N_{\text{total}}\times D}$ be the learnable content embeddings representing global semantic patterns, and let $M_{\text{vid}}\in {\mathbb{R}}^{L_{v}\times D}$ denote the video portion of the encoder output (Section Decoupled feature encoding for unbiased perception). The multi-scale Gaussian pooling module extracts instance-specific context $P_{\text{gauss}}\in {\mathbb{R}}^{N_{\text{total}}\times D}$ from *M*_vid_ rather than from the raw input features, ensuring that the pooled representations incorporate the cross-modal alignment learned by the encoder. The initialized content query $q_{i}^{\text{content}}$ at the *i*-th position is calculated via a residual connection:


$$\begin{array}{c} {𝐪}_{i}^{\text{content}}=\mathrm{LayerNorm}({𝐜}_{i}+{𝐩}_{\text{gauss},i}) \end{array}$$ (2)

where ${𝐜}_{i}\in C$ and ${𝐩}_{\text{gauss},i}$ is the *i*-th output of the Gaussian pooling applied to *M*_vid_. This residual fusion allows the model to dynamically adapt to the encoded video context while preserving stable semantic anchors.

Meanwhile, to provide explicit temporal guidance, the module directly utilizes the Gaussian centers to initialize the positional part of the queries. The positional query $q_{i}^{\text{pos}}$ encodes the center $\mu_{i}$ of the corresponding Gaussian kernel using a sinusoidal positional encoding function PE(·):


$$\begin{array}{c} {𝐪}_{i}^{\text{pos}}=PE(\mu_{i}),\;\mu_{i}=\mathrm{sigmoid}(\mu_{i}^{\text{raw}}) \end{array}$$ (3)

where $\mu_{i}^{\text{raw}}$ is the unconstrained learnable parameter and sigmoid(·) constrains the center to the valid temporal range [0, 1].

By passing this set of enhanced queries $𝒬=\{(q_{i}^{\text{content}},q_{i}^{\text{pos}})\}$ to the decoder, spatiotemporal inductive biases are injected. This strategy encourages each query to initiate its search from a semantically relevant and temporally reasonable position, which may improve scale-specific localization behavior.

### Decoupled feature encoding for unbiased perception

A key design choice in our architecture is the strict separation between feature encoding and query-guided decoding. The encoder processes only the video and text inputs without access to any retrieval-specific query priors:


$$\begin{array}{c} S=[{𝐗}_{\text{vid}}⊕{𝐗}_{\text{txt}}],\;M=ℰ(S,\text{Mask},{𝒫}_{\text{pos}}) \end{array}$$ (4)

where ⊕ denotes concatenation along the temporal dimension, and ℰ denotes the Transformer encoder. We denote the video portion of the encoded representation as $M_{\text{vid}}\in {\mathbb{R}}^{L_{v}\times D}$.

This design follows the established DETR encoder-decoder paradigm [22], where the encoder contextualizes the input features and the decoder uses learned queries to produce task-specific set predictions. In our context, this separation serves two purposes:

(1) **Query-agnostic cross-modal representation.** By excluding query priors from the encoder input, the self-attention mechanism learns video-text alignments without being biased toward specific temporal locations. This makes *M* a query-agnostic representation of the video-text pair.
(2) **Clean context for Gaussian pooling.** Because Gaussian pooling extracts context from the encoder output *M*_vid_ (Section Dynamic context-aware query initialization), the quality of the pooled representations depends on *M* being free from query-induced artifacts. If query priors were injected during encoding, the Gaussian pooling would inherit and amplify these biases, potentially degrading localization for events at scales not well-represented by the initial queries.

We emphasize that Gaussian pooling operates as a *read-only* aggregation over the completed encoder output: it extracts weighted features from *M*_vid_ but does not affect the encoder’s internal self-attention computation. This design keeps the Gaussian prior injection outside the encoder and preserves the standard DETR-style separation between encoding and decoding.

## Experimental results and analysis

The main experiments of this paper are conducted on QVHighlights, an authoritative benchmark dataset in the field of video moment retrieval. QVHighlights is a highly challenging, large-scale multimodal dataset built from over 10,000 YouTube videos, covering a wide range of topics from everyday activities and travel in lifestyle vlog videos to social and political activities in news videos. This dataset provides precise temporal boundary annotations for each natural language query. The dataset comprises 7,218 training, 1,550 validation, and 1,542 test queries.

Following the standard evaluation protocol, we report scale-specific metrics (Short mAP, Middle mAP, Long mAP) in addition to overall recall (R1@0.5, R1@0.7) and Full mAP. The event-length categories are defined by the ground-truth window duration: Short (≤10s), Middle (10–30s], and Long (>30s). In the validation set, 429 queries (27.7%) contain at least one short-duration relevant window, 957 (61.7%) contain at least one middle-duration window, and 574 (37.0%) contain at least one long-duration window. The percentages sum to more than 100% because some queries have multiple relevant windows of different durations. Short-event localization is particularly challenging under the QVHighlights evaluation protocol: the 2-second clip length limits the temporal resolution for events lasting only a few seconds, and short events provide fewer salient visual cues for the model to anchor on.

### Implementation details

All models use pre-extracted visual features from SlowFast [23] (2,304-dimensional) and CLIP ViT-B/32 visual encoder [24] (512-dimensional), concatenated to form 2,816-dimensional video features, plus 2-dimensional temporal embedding features (per-clip start/end boundaries), yielding an effective video input dimension of 2,818. Text features are extracted using the CLIP text encoder (512-dimensional, last hidden state, max 32 tokens). All features are L2-normalized before input. The feature extractors are frozen during training.

The Transformer encoder and decoder each consist of 2 layers with a hidden dimension of 256, feedforward dimension of 1,024, and 8 attention heads. Input dropout is 0.5 and Transformer dropout is 0.1. The model is trained with the AdamW optimizer (learning rate $1\times 10^{-4}$, weight decay $1\times 10^{-4}$, gradient clipping at 0.1) for 200 epochs with a batch size of 32. The learning rate schedule is StepLR, though the drop epoch (400) exceeds the training duration, effectively keeping the learning rate constant. The span prediction loss is L1 regression on (center, width) with coefficient 10, combined with a GIoU loss (coefficient 1) and a binary cross-entropy classification loss (coefficient 4). A saliency hinge loss (coefficient 1.0, margin 0.2) is applied to the top decoder layer only. Hungarian matching uses the same loss weights. Text positional encoding and contrastive alignment loss are disabled by default.

### Experiments and overall performance analysis

This section systematically evaluates Gaussian-DETR on the QVHighlights validation split and compares it with the baseline model Moment-DETR. The main comparison uses 15 matched random seeds and reports the mean and standard deviation. For each seed and method, the checkpoint used in the main comparison was selected according to validation R1@0.5. The core objective of this paper is to evaluate whether explicit temporal priors provide scale-specific benefits, especially for long-duration events, while making the trade-off with overall recall explicit.

#### Overall performance comparison.

To assess whether the observed differences are robust across random seeds, we performed paired statistical analysis over 15 matched seeds (Table 2). For each metric, we computed the per-seed difference between Gaussian-DETR and Moment-DETR and report the 95% confidence interval of the paired mean difference using Student’s *t* distribution. Gaussian-DETR achieved a mean Long mAP improvement of +1.98 points, with a 95% confidence interval of [+0.53, + 3.43] and a paired *t*-test *p* = 0.0111, providing the strongest statistical support for the proposed temporal prior. For R1@0.5 and R1@0.7, the paired differences are close to zero with confidence intervals that span both positive and negative values, indicating no significant change in overall recall. Short mAP shows a modest positive trend (+0.24), though its confidence interval also overlaps zero.

**Table 2:** Paired statistical analysis over 15 random seeds on QVHighlights. Differences are computed as Gaussian-DETR minus Moment-DETR.

Metric	Mean difference	95% CI	Paired *t*-test *p*	Cohen’s *d*
R1@0.5	−0.59	[−1.58,+0.40]	0.2230	−0.33
R1@0.7	−0.17	[−1.46,+1.11]	0.7755	−0.08
Long mAP	+1.98	[+0.53, +3.43]	0.0111	+0.76
Short mAP	+0.24	[−0.02,+0.49]	0.0639	+0.52

Tables 3 and 4 present the performance comparison between Gaussian-DETR and Moment-DETR across primary evaluation metrics, including R1@0.5, R1@0.7, and Short/Long mAP categorized by event temporal length. The optimal epochs for different evaluation metrics are not perfectly aligned. This phenomenon also exists in the baseline model, indicating that when optimizing the overall recall rate further, the model might sacrifice some specialized modeling capability for extreme temporal scales. To ensure a consistent comparison protocol and avoid selecting different checkpoints for individual sub-metrics, all main results are reported using the checkpoints selected by validation R1@0.5. S1 and S2 Appendices provide diagnostic best-per-metric and training-dynamics analyses that characterize metric-specific convergence behavior.

**Table 3:** Gaussian-DETR validation results. All metrics are percentages; higher is better.

Seed	R1@0.5	R1@0.7	Long mAP	Short mAP
1	52.06	34.97	41.68	3.42
7	54.45	34.71	43.22	3.27
13	57.23	37.16	44.92	3.58
37	53.10	33.10	41.14	3.69
42	54.90	34.26	41.06	3.74
99	52.45	34.65	40.94	3.28
123	54.45	36.52	42.39	3.87
456	54.45	37.03	43.64	3.91
777	52.39	33.74	39.34	4.32
1024	53.94	37.10	42.04	3.38
2022	55.61	37.74	45.32	3.60
2023	55.48	36.71	42.34	4.48
2024	56.45	38.52	44.34	4.20
2025	56.19	38.65	44.28	4.17
2048	51.87	35.74	43.51	3.61
Mean	54.33	36.04	42.68	3.77
Std	1.69	1.73	1.69	0.38

**Table 4:** Moment-DETR validation results. All metrics are percentages; higher is better.

Seed	R1@0.5	R1@0.7	Long mAP	Short mAP
1	54.71	36.26	40.99	3.22
7	55.03	37.03	39.83	3.40
13	55.03	37.35	41.30	3.12
37	53.16	35.35	39.21	3.21
42	55.87	37.42	42.00	3.48
99	54.39	36.71	40.78	3.54
123	56.39	38.19	43.97	3.11
456	53.10	34.19	39.88	3.80
777	54.52	36.00	41.36	3.34
1024	55.03	35.61	41.95	3.25
2022	54.84	34.65	38.72	4.01
2023	55.55	34.65	39.62	3.73
2024	54.90	34.90	39.95	3.76
2025	55.23	37.48	38.53	3.75
2048	56.13	37.42	42.37	4.26
Mean	54.93	36.21	40.70	3.53
Std	0.92	1.27	1.50	0.34

In terms of overall recall, the paired analysis is statistically inconclusive. The paired mean differences for R1@0.5 and R1@0.7 are small and negative, and both confidence intervals include zero. We therefore interpret the overall-recall effect as statistically inconclusive, with a small negative point estimate under this protocol.

**Fig 2:**
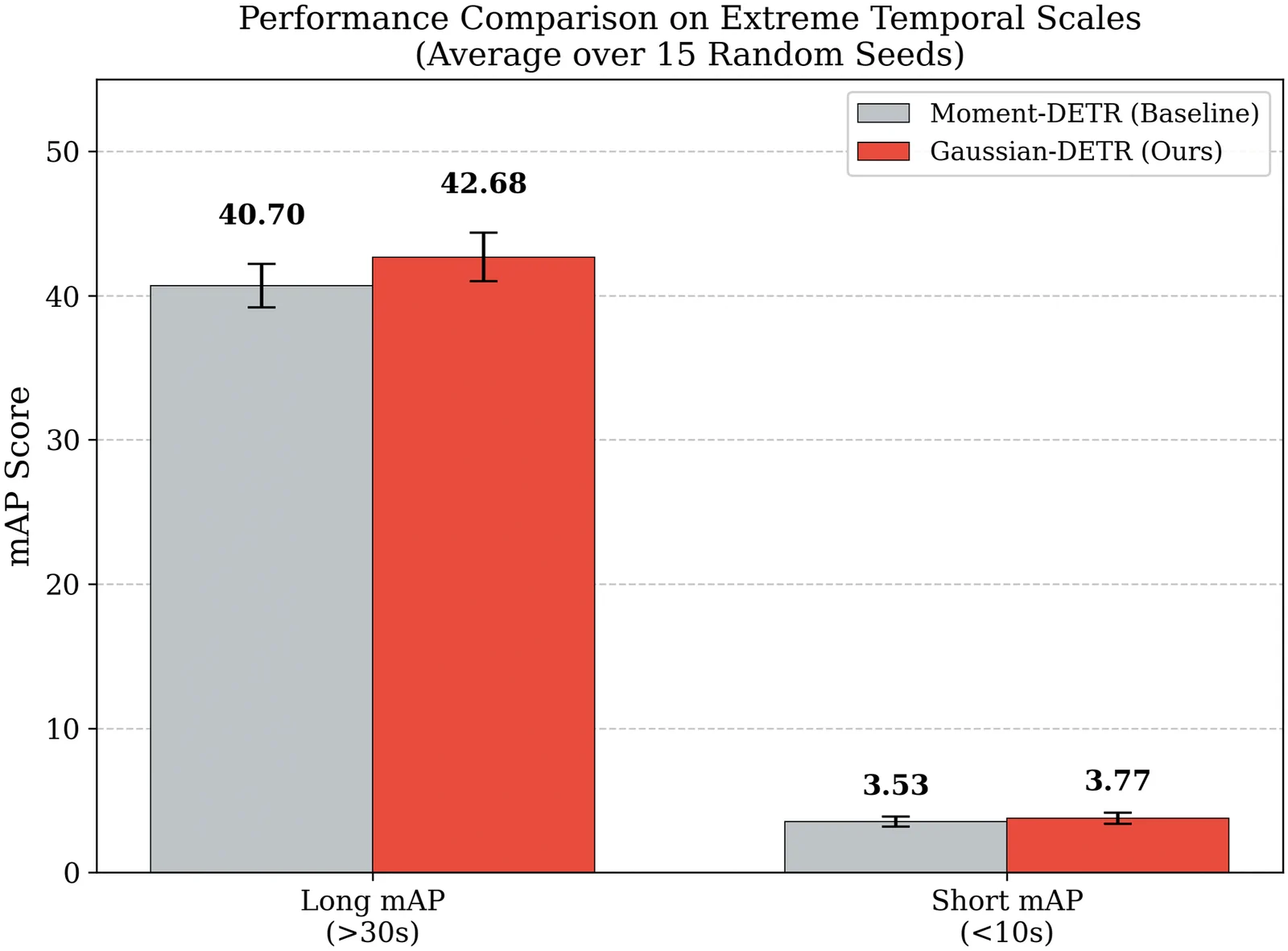
Comparison of Gaussian-DETR and Moment-DETR on QVHighlights under different random seeds.

**Fig 3:**
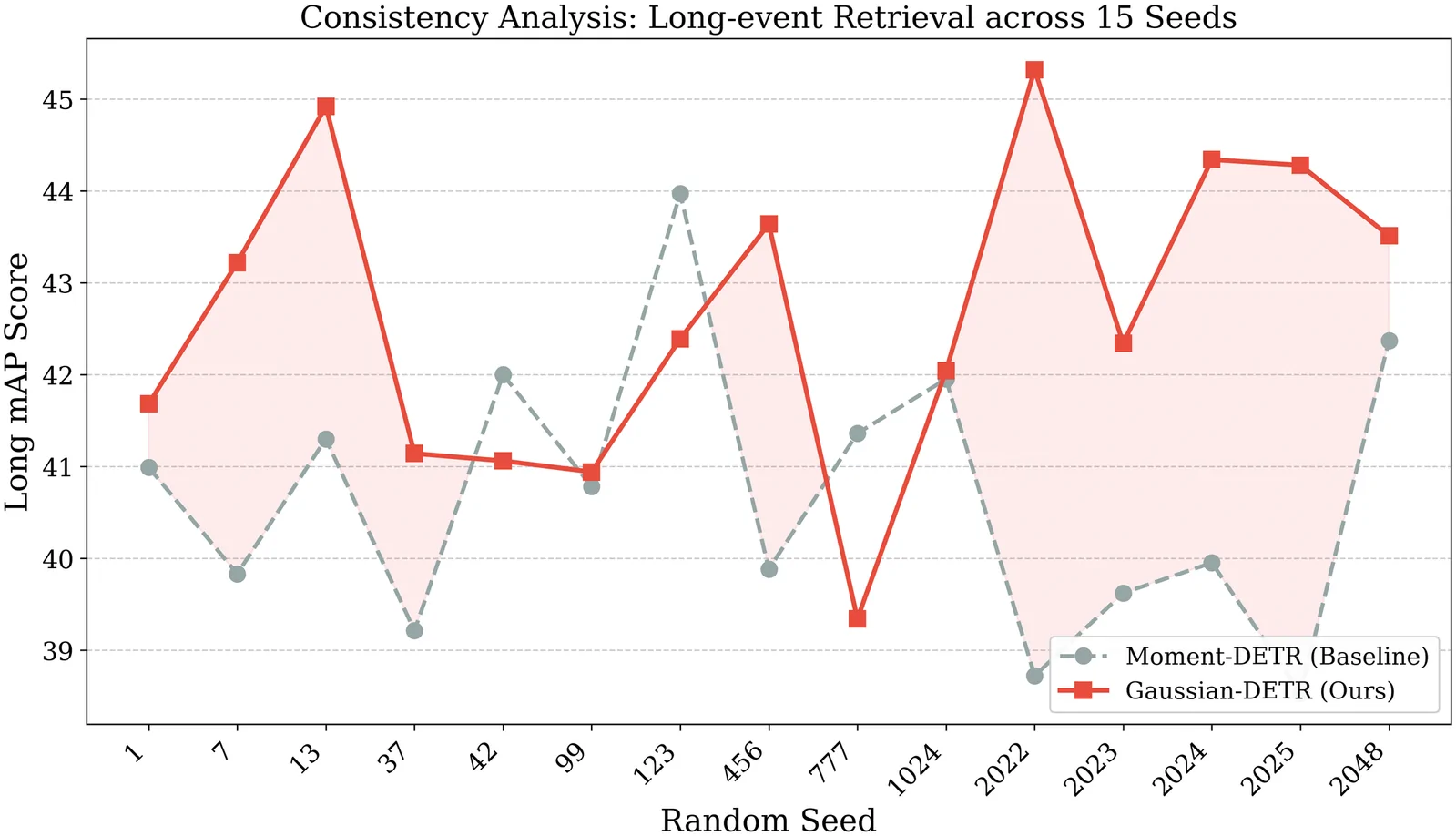
Consistency analysis of Long mAP under different random seeds.

On extreme temporal scales, Gaussian-DETR shows its clearest advantage on long-event retrieval. Compared with Moment-DETR, Long mAP increases from 40.70 to 42.68 on average across 15 seeds. The paired confidence interval is fully positive, indicating that this is the most robust scale-specific improvement in our main comparison. Short mAP increases from 3.53 to 3.77 on average, but the paired confidence interval overlaps zero and the absolute values remain low. We therefore report the short-event result as a modest positive trend. The low absolute Short mAP values reflect the inherent difficulty of short-event localization under the QVHighlights evaluation protocol: with a clip length of 2 seconds, a 5-second event spans only 2–3 clips, providing limited temporal resolution for precise boundary regression. Additionally, short events constitute only 27.7% of validation queries, further constraining the signal available for learning short-duration patterns. As shown in Fig 2, Gaussian-DETR provides a scale-specific benefit for long-duration events; the consistency analysis in Fig 3 further supports the long-event retrieval trend across random seeds.

From a practical perspective, the increase in Long mAP from 40.70 to 42.68 (+1.98 points, approximately 4.9% relative) indicates that Gaussian-DETR provides its clearest demonstrated benefit for long-duration moment retrieval. This setting is relevant to queries describing extended activities or context-dependent events, for which successful localization requires preserving evidence over a broad temporal span rather than relying on a single salient short interval. Together with the statistically inconclusive differences in R1@0.5 and R1@0.7 and the low absolute Short mAP, these results characterize Gaussian-DETR as providing a scale-specific gain, with the clearest support on Long mAP under the evaluated protocol.

#### Analysis of the role of multi-scale Gaussian priors.

Traditional Transformer-based temporal localization models typically rely on implicitly learned query representations, where the temporal attention range is adaptively determined entirely by the attention mechanism. This approach performs well on medium-length events but can exhibit attention-range misalignment—such as an excessively broad or narrow attention range—in scenarios involving extremely long or short events.

By introducing explicit multi-scale Gaussian temporal priors, Gaussian-DETR provides each query with a learnable temporal center (μ) and coverage range (σ), introducing scale-aware initialization during the decoding stage:

Coarse-grained Gaussian queries (Coarse Scale) have a large temporal coverage range and help aggregate broader context in long videos, reducing context dilution in long events.

Fine-grained Gaussian queries (Fine Scale) are densely distributed along the temporal axis, providing local temporal probes for short-duration segments. This design increases local sampling density, which is consistent with the short-event trend observed in the ablation results.

This explicit temporal structural constraint provides a plausible basis for the observed scale-specific behavior: the main 15-seed analysis supports Long mAP improvement, while Short mAP shows only a modest positive trend.

### Ablation studies and mechanistic analysis

This section presents four component-level ablation studies that isolate the contributions of individual design choices in Gaussian-DETR. All ablations share a fixed five-seed subset (42, 2022, 2023, 2024, and 2025) to enable controlled architectural comparisons.

#### Is the increase in the number of queries the primary reason for performance differences?.

**Fig 4:**
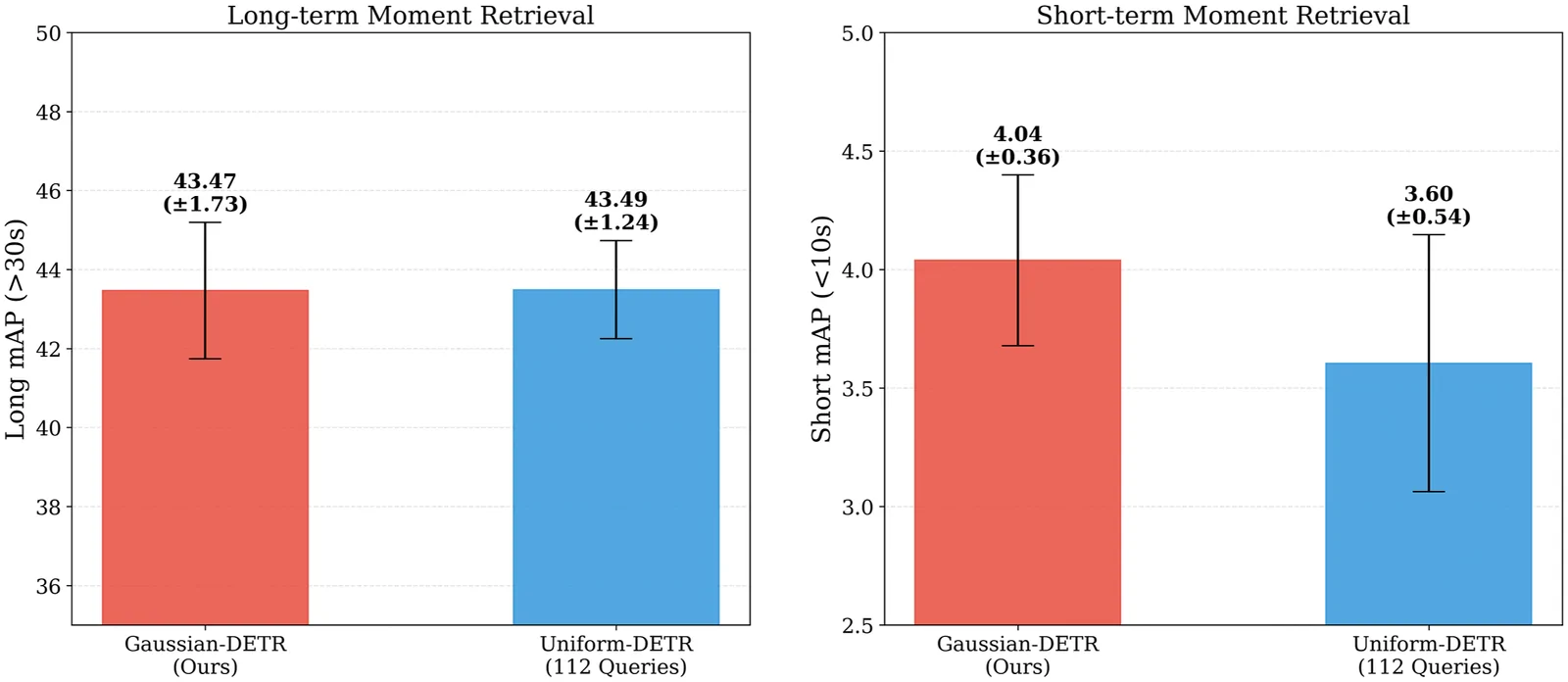
Performance comparison between Gaussian-DETR and Uniform-DETR on extreme temporal scales.

To investigate how much of the observed behavior can be attributed to query quantity, this experiment constructs a Uniform-DETR control baseline. This model maintains the exact same total number of queries as Gaussian-DETR but removes the explicit structural priors provided by Gaussian pooling during the initialization stage, retaining only static learnable semantic content. This setting helps separate the “quantity” and “structure” variables. Relevant results are detailed in Table 5 and Fig 4.

**Table 5:** Uniform-DETR experimental results on the fixed five-seed ablation subset. All metrics are percentages; higher is better.

Seed	R1@0.5	R1@0.7	Long mAP	Short mAP
42	52.32	35.16	42.13	3.78
2022	53.23	36.06	44.13	3.43
2023	54.45	34.97	44.06	3.09
2024	55.61	36.32	44.90	4.46
2025	52.71	32.39	42.22	3.26
Mean	53.66	34.98	43.49	3.60
Std	1.34	1.56	1.24	0.54

Experimental Results and Analysis: Within the fixed five-seed ablation subset, the results reveal a trade-off between query quantity and query structure. Uniform-DETR achieves Long mAP (43.49 ± 1.24) comparable to the Gaussian-DETR full-model reference (43.47 ± 1.73), indicating that a high density of unconstrained queries can cover long-span events even without explicit positional guidance. However, this coverage comes with lower overall-recall performance in the same ablation subset: Uniform-DETR’s R1@0.5 (53.66 ± 1.34) falls below Gaussian-DETR (55.73 ± 0.61), suggesting reduced overall-recall stability across random seeds.

These component-level results suggest that simply increasing query count provides a mixed effect. While the additional queries can improve coverage for long events, the lack of explicit temporal anchors may increase query competition during decoding. The multi-scale Gaussian prior is therefore best interpreted as a trade-off mechanism: it combines dense query coverage with temporal structure, rather than proving that the Gaussian prior alone determines Long mAP.

#### Is the Gaussian distribution superior to unstructured global context?.

**Fig 5:**
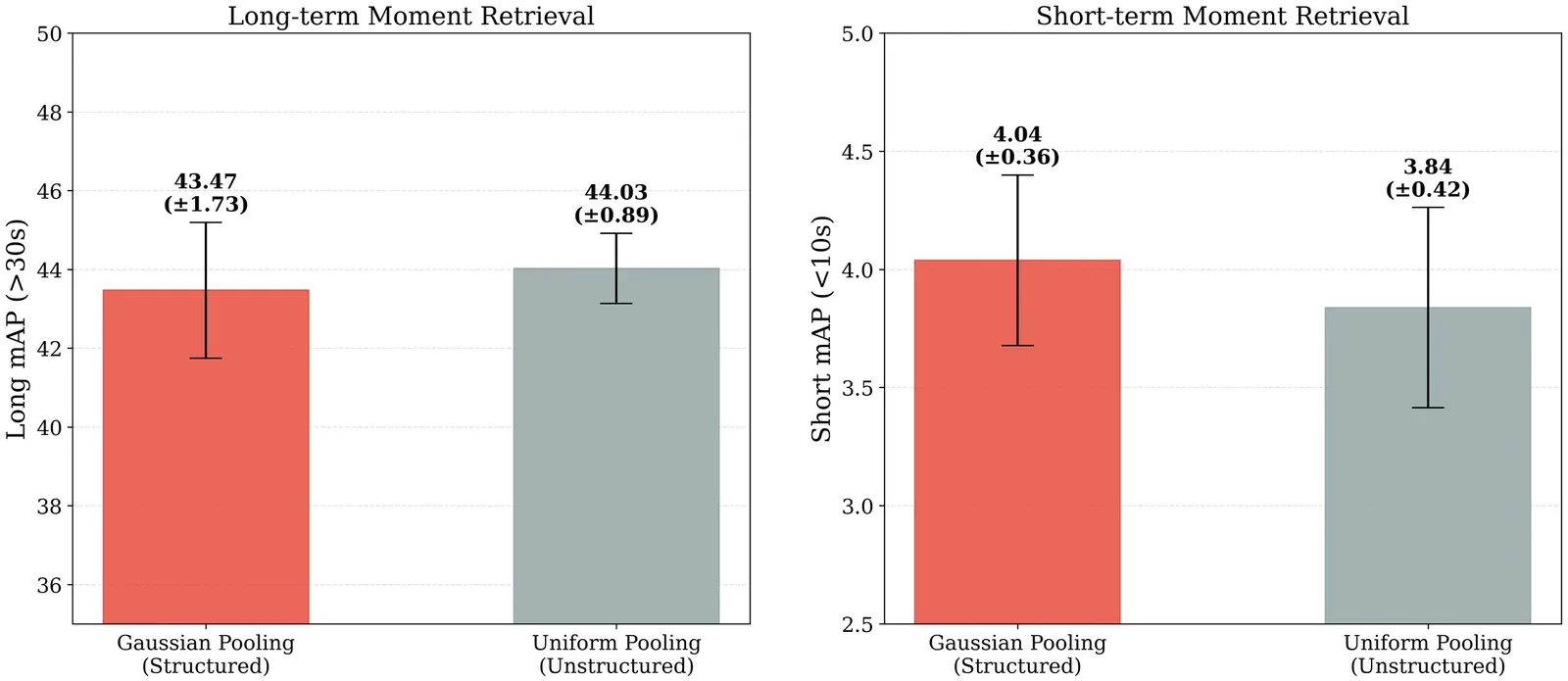
Performance comparison between structured Gaussian pooling and unstructured global pooling.

Experimental Setup: To evaluate the role of explicit temporal structures in context aggregation, this section replaces the Gaussian-weighted pooling module with standard Masked Global Average Pooling to construct an unstructured pooling variant (Uniform Pooling). This variant assigns uniform weights to all frames along the temporal dimension to aggregate the visual features of the entire video sequence, simulating traditional context modeling approaches that lack temporal positional priors. Relevant experimental results are shown in Table 6 and Fig 5.

**Table 6:** Experimental results of unstructured global pooling (Uniform Pooling) on the fixed five-seed ablation subset. All metrics are percentages; higher is better.

Seed	R1@0.5	R1@0.7	Long mAP	Short mAP
42	53.87	35.10	43.65	4.19
2022	54.00	35.23	44.41	3.97
2023	54.71	36.97	44.81	3.97
2024	53.61	34.77	42.64	3.10
2025	53.81	34.19	44.62	3.96
Mean	54.00	35.25	44.03	3.84
Std	0.42	1.04	0.89	0.42

Experiments and Result Analysis: Within the fixed five-seed ablation subset, global average pooling achieves Long mAP (44.03 ± 0.89) slightly higher than the Gaussian-DETR full-model reference (43.47 ± 1.73). Since long-term events span most of the video, their features are distributed broadly along the temporal axis; uniform aggregation over all frames naturally captures this coarse-grained context. This component-level finding highlights that for long-event retrieval specifically, unstructured global context can be competitive with structured pooling.

However, the global pooling variant shows lower R1@0.5 (54.00 ± 0.42 vs. 55.73 ± 0.61) and Short mAP (3.84 ± 0.42 vs. 4.04 ± 0.36) compared to Gaussian-DETR in the same ablation subset. The uniform averaging operation assigns equal weight to both relevant and irrelevant frames, which can dilute local temporal signals needed for precise boundary regression. The multi-scale Gaussian pooling instead concentrates weight around each query’s temporal center while still allowing context through the kernel width, suggesting a better balance between long-range coverage and local specificity.

#### Is the fine-grained scale necessary for short-term actions?.

**Fig 6:**
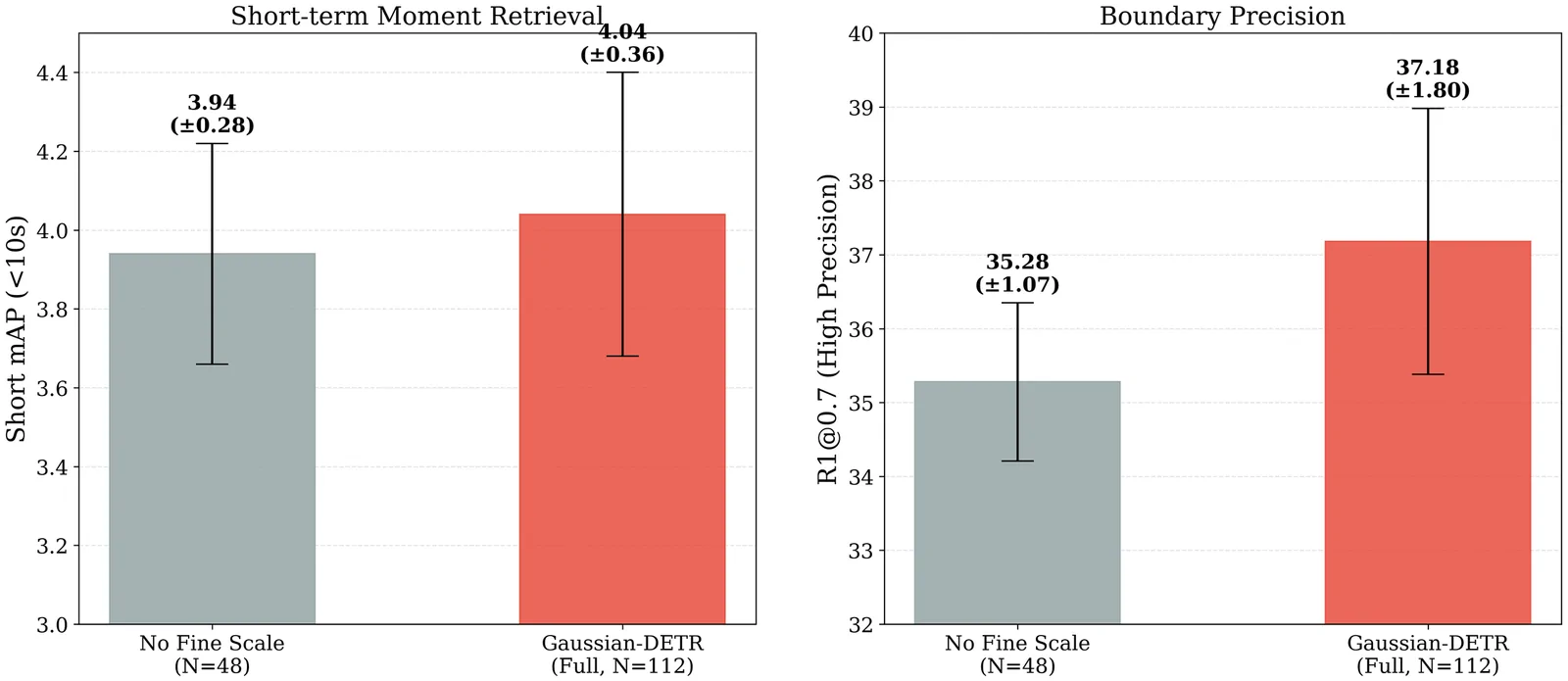
Impact of fine-grained scale on short-term actions and local temporal sampling.

To analyze the contribution of the fine-grained layer in the multi-scale design, this section constructs two ablation variants. The first removes the fine-grained layer and reduces total query count from 112 to 48 (Original Ablation). The second removes the fine-grained layer while redistributing queries to maintain 112 total (Fixed-Query Ablation), isolating the contribution of scale structure from query quantity. Relevant experimental results are shown in Table 7 and Fig 6.

**Table 7:** Ablation study of fine-grained scale on the fixed five-seed ablation subset. Original ablation removes Fine (*N*_1_ = 64, total 48 queries). Fixed-query ablation redistributes to Medium (80) + Coarse (32), keeping total at 112. All metrics are percentages; higher is better. The full-model reference is the five-seed ablation-subset mean and standard deviation.

Configuration	Queries	R1@0.5	R1@0.7	Long mAP	Short mAP
*Original Ablation (reduced query count)*					
w/o Fine	48	55.22 ± 0.69	35.28 ± 1.07	41.33 ± 0.81	3.94 ± 0.28
*Fixed-Query Ablation (constant query count)*					
w/o Fine	112	54.52 ± 0.90	36.14 ± 0.89	43.21 ± 1.18	3.68 ± 0.45
*Full Model (reference)*					
Full (64/32/16)	112	55.73 ± 0.61	37.18 ± 1.80	43.47 ± 1.73	4.04 ± 0.36

Experimental Results and Analysis: Within the fixed five-seed ablation subset, removing fine-grained queries directly causes a decline in performance across all metrics. In the original ablation (total 48 queries), Short mAP decreases from 4.04 to 3.94 and R1@0.5 decreases from 55.73 to 55.22. However, this comparison conflates two effects: the loss of fine-grained temporal sampling and the reduction in total query count.

To isolate the contribution of hierarchical scale structure, the fixed-query ablation maintains 112 total queries by redistributing the fine-grained slots to medium (80) and coarse (32) scales. Even with the same query budget, removing the fine-grained level yields a 1.21-point lower R1@0.5 (55.73 vs. 54.52) and a 0.26-point lower Long mAP (43.47 vs. 43.21) within this ablation setting. This suggests that the fine-grained layer contributes useful high-frequency temporal sampling that is not fully compensated by simply increasing medium and coarse queries. The Short mAP decline (4.04 vs. 3.68, −8.9%) is component-level evidence for local temporal sampling, while the main 15-seed results keep the short-event conclusion limited to a modest trend.

This performance drop is consistent with a mismatch between query scale and event duration. Short actions occupy few clips, whereas medium and coarse queries use wider Gaussian spans. When only wider queries are available, local evidence for a short target can be averaged with surrounding context, weakening boundary cues.

By introducing high-density, narrow-bandwidth temporal probes, fine-grained queries increase the density of local temporal hypotheses available to the decoder. This mechanism may help preserve local responses for fleeting actions, although the main 15-seed results show that Short mAP remains low in absolute terms and only exhibits a modest trend.

#### Sensitivity to the fine-scale kernel-width scaling factor.

**Fig 7:**
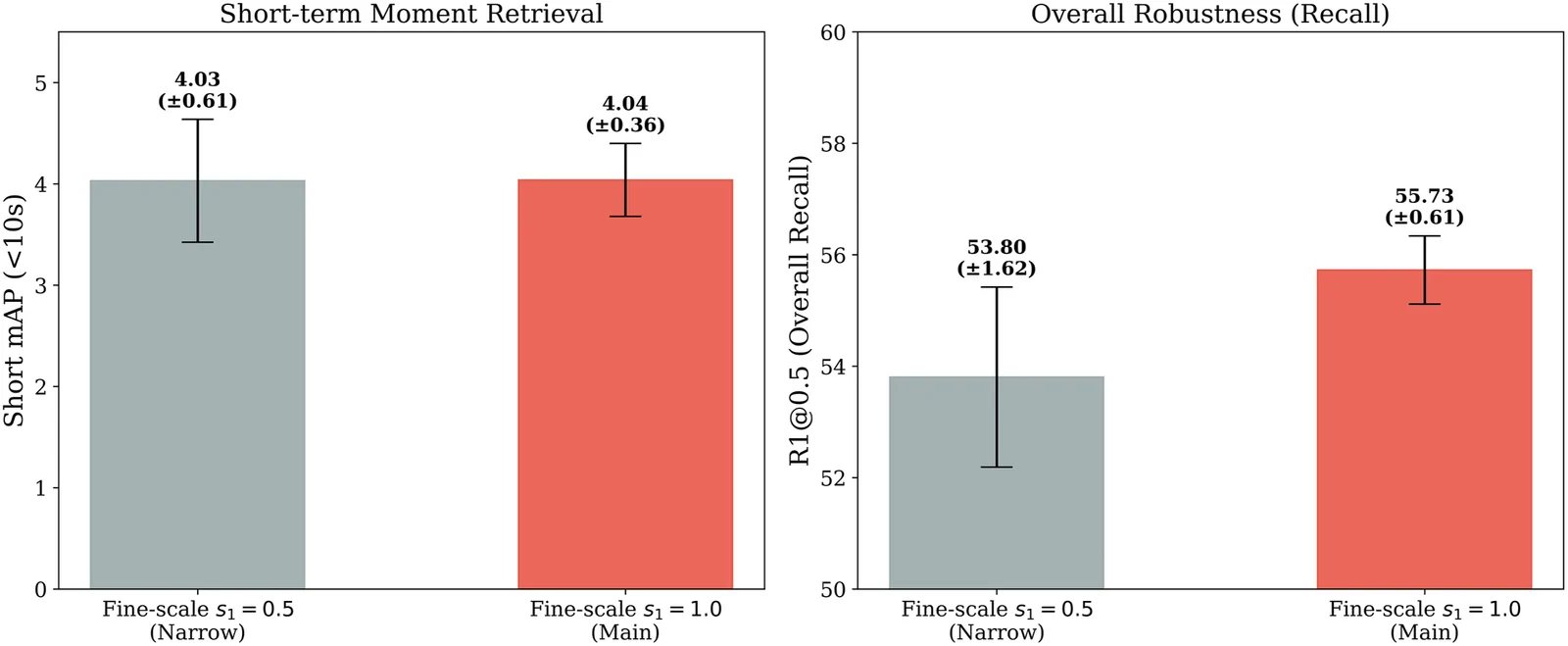
Sensitivity to the fine-scale kernel-width scaling factor under two initialization settings.

Experimental Setup: To examine the effect of fine-scale temporal prior width, this section reports a two-setting sensitivity check on the fine-scale kernel-width scaling factor *s*_1_. According to the initialization rule $\sigma_{l,i}=s_{l}/N_{l}$, the main configuration uses *s*_1_ = 1.0, whereas the narrower variant uses *s*_1_ = 0.5; the medium- and coarse-scale settings remain unchanged. This comparison evaluates the effect of narrowing the fine-scale initialization while keeping the medium- and coarse-scale settings fixed. Relevant results are shown in Table 8 and Fig 7.

**Table 8:** Results for the narrower fine-scale kernel-width initialization setting *s*_1_ = 0.5 on the fixed five-seed ablation subset. The main *s*_1_ = 1.0 reference is reported in the Full Model row of Table 7; the medium- and coarse-scale settings remain unchanged. All metrics are percentages; higher is better.

Seed	R1@0.5	R1@0.7	Long mAP	Short mAP
42	55.48	36.71	43.16	3.68
2022	51.87	33.68	40.11	3.79
2023	52.90	35.10	42.32	3.82
2024	53.29	33.29	40.11	5.11
2025	55.48	37.23	42.96	3.75
Mean	53.80	35.20	41.73	4.03
Std	1.62	1.76	1.51	0.61

Experimental Results and Analysis: Compared with the full-model reference using *s*_1_ = 1.0 in Table 7, the *s*_1_ = 0.5 variant yields lower R1@0.5 and Long mAP, without a clear benefit for short-event retrieval. These results indicate sensitivity to a narrower fine-scale initialization, particularly for R1@0.5 and Long mAP. Because the Gaussian spans remain learnable during training, this result characterizes sensitivity to the initialization width under the tested two-setting comparison.

Taken together, the main comparison and ablation studies indicate that Gaussian-DETR is best interpreted as a scale-aware query initialization mechanism whose clearest empirical benefit is long-event retrieval. These ablations characterize the empirical trade-offs among query quantity, temporal structure, and pooling strategy, while more detailed causal analysis of the underlying attention dynamics is left for future work.

### Architectural compatibility with QD-DETR

To examine whether the proposed Gaussian prior can be integrated into another DETR-like architecture, we apply it to QD-DETR [9] while retaining the original feature setting and encoder interaction mechanisms. This experiment evaluates compatibility with one additional DETR-like architecture rather than broad architectural generalization. The modification is limited to decoder-query construction: multi-scale Gaussian pooling is applied to the completed encoder output, and the pooled representations are used for context-aware query initialization.

Table 9 reports both same-protocol QD-DETR comparisons and literature-reported results from recent methods. The lower block provides contextual reference only, because these studies use different feature settings, training protocols, and metric reporting.

**Table 9:** Performance comparison on QVHighlights. All metrics are percentages; higher is better. Upper block: same QD-DETR base architecture and SlowFast+CLIP features. Lower block: literature-reported results under different settings. For literature rows, “Full” denotes the reported overall/average mAP; “–” indicates an unavailable or unreported metric.

Method	Features / Notes	R1@0.5	Short	Middle	Long	Full
*Same protocol (QD-DETR base, SlowFast+CLIP)*						
QD-DETR	baseline	61.22	7.77	43.10	47.44	41.00
LAD (w/o MM)	+ length cond.	–	11.01	45.53	50.76	44.48
Gaussian-QD-DETR	+ Gaussian prior	64.45	10.91	47.45	49.05	44.49
*Literature-reported / recent methods (different settings)*						
LAD (w/ MM) [11]	+ MomentMix	63.62	–	–	–	46.61
TR-DETR [8]	reported test split	64.66	–	–	–	42.62
UniVTG [25]	reported test split	58.86	–	–	–	35.47
EaTR [26]	reported val split	61.36	–	–	–	41.74
CG-DETR [27]	reported test split	65.4	–	–	–	42.9

Under the same protocol, Gaussian-QD-DETR improves over the vanilla QD-DETR baseline across all reported metrics, including R1@0.5 (61.22→64.45), Full mAP (41.00→44.49), and Short mAP (7.77→10.91). The literature rows place this result in the range of recent temporal grounding methods, while the protocol differences preclude a strict state-of-the-art ranking.

### Auxiliary evaluation on Charades-STA

In addition to QVHighlights, we include Charades-STA as a secondary benchmark [1]. Table 10 reports this auxiliary evaluation. Both Moment-DETR and Gaussian-DETR are trained and evaluated with the same available SlowFast+CLIP feature pipeline, so the result is interpreted as a controlled within-pipeline comparison.

**Table 10:** Auxiliary evaluation on Charades-STA. Results are reported over two random seeds (2022 and 2023) under the same available SlowFast+CLIP reproduction pipeline. All metrics are percentages; higher is better.

Method	R1@0.5	R1@0.7	Notes
Moment-DETR	37.65±0.13	20.42±0.47	Charades-trained baseline
Gaussian-DETR	37.50±0.79	19.89±0.11	Charades-trained Gaussian prior
Difference	−0.15	−0.53	Gaussian-DETR minus Moment-DETR

Under this limited two-seed setting, Gaussian-DETR shows no clear advantage over Moment-DETR on Charades-STA, with small observed differences of −0.15 points in R1@0.5 and −0.53 points in R1@0.7. This auxiliary evaluation provides an additional within-pipeline benchmark check; the strongest empirical support remains the Long mAP improvement on QVHighlights.

### Computational complexity analysis

To assess the practical overhead introduced by the Gaussian prior module, we compare parameter counts, multiply-accumulate operations (MACs), and inference latency between Moment-DETR and Gaussian-DETR (Table 11). MACs are estimated analytically for a single forward pass with input length *L*_vid_ = 75 clips and *L*_txt_ = 32 tokens. Latency is measured on an NVIDIA H800 GPU with batch size 32, averaged over 100 iterations after 10 warm-up iterations.

**Table 11:** Computational complexity comparison.

Model	Params	MACs	Latency (ms)
Moment-DETR (10 queries)	4.82M	0.31G	3.2
Gaussian-DETR (112 queries)	4.85M	0.50G	4.9
Overhead	+0.6%	+62%	+1.7

Gaussian-DETR introduces a parameter overhead of only +0.6% (26,848 additional parameters), mainly due to the expansion of the query content embeddings from 10 to 112 slots, with smaller contributions from the Gaussian pooling parameters and query normalization. The MAC overhead is + 62%, corresponding to approximately 0.19G additional MACs at the model level. This increase mainly reflects query-dependent decoder computation: decoder cross-attention scales approximately linearly with the number of queries, whereas decoder self-attention scales quadratically through query-query interactions. In contrast, Gaussian pooling itself is a lightweight weighted aggregation over temporal clips. The module therefore introduces a measurable computational cost, but the measured model-level latency remains below 5 ms per batch-size-32 forward pass on the tested GPU.

**Practical deployment considerations.** Although the Gaussian prior module introduces only a small parameter increase, expanding the decoder query set from 10 to 112 produces a measurable computational cost. Under the tested setting, the model-level forward latency remains below 5 ms on an NVIDIA H800 GPU with a batch size of 32. The measurement covers model-level forward latency with pre-extracted features; video decoding, visual and textual feature extraction, and data-transfer overhead are outside this timing protocol. In latency-sensitive or resource-constrained deployments, the query budget may need to be adjusted according to the target hardware, batch size, input sequence length, and application-level latency requirements.

### Summary

Through quantitative experiments and ablation analyses, this section characterizes Gaussian-DETR as a scale-specific temporal prior for query initialization. Its most consistent benefit appears in long-event retrieval, and the ablations indicate that structured Gaussian queries provide a better balance than simply increasing query count or using unstructured global pooling. These controls suggest that the benefit comes from coupling long-event coverage with local temporal specificity rather than from query density alone.

The explicit multi-scale Gaussian prior introduced in this paper is best understood as a structured trade-off between temporal coverage and localization specificity. Fine-grained temporal probes increase local sampling density, while wider Gaussian kernels aggregate long-range context. The combination is best characterized as a scale-specific prior that biases decoder queries toward long-duration temporal hypotheses.

Experimental results integrating this prior module into the QD-DETR architecture further support compatibility beyond the Moment-DETR baseline. Because this strategy focuses on the initial construction stage of decoder queries while leaving the framework’s underlying feature interaction and loss calculation logic unchanged, it can be studied as a parameter-efficient scale-aware initialization component for DETR-like video moment retrieval models.

## Discussion

### Mechanism decoupling in multi-scale modeling

An inherent tension exists between temporal span and resolution in video understanding: capturing global semantics requires a large temporal receptive field, while precise boundary estimation benefits from high temporal resolution. Gaussian-DETR addresses this tension at the query-initialization stage by assigning decoder queries to temporal hypotheses with different centers and spans. Coarse-grained queries provide broader temporal coverage, whereas fine-grained queries provide denser local sampling. This structure is consistent with the observed trade-off in our experiments: long-event retrieval benefits most consistently, while short-event localization remains difficult under the current QVHighlights protocol.

### Convergence patterns of temporal feature learning

The training-dynamics analysis in S2 Appendix shows that overall recall and scale-specific metrics do not necessarily peak at the same epoch. In particular, the epoch selected by R1@0.5 can differ from the epoch that gives the highest Long mAP. This pattern indicates that overall retrieval quality and scale-specific temporal modeling follow partially different optimization trajectories. The explicit Gaussian prior provides structured temporal hypotheses throughout training, which may help preserve long-span candidates even when the checkpoint is selected by an overall-recall criterion. This motivates the separation between the main R1@0.5-selected comparison and the diagnostic best-per-metric analysis in S1 Appendix.

## Conclusion

This paper proposes Gaussian-DETR, a video temporal localization framework that initializes Transformer decoder queries via explicit multi-scale Gaussian temporal priors. Distinct from existing end-to-end models relying on implicit queries, this method directly parameterizes temporal center and scale information and injects them into the decoding hypothesis space, providing explicit temporal structural guidance. Combined with Gaussian pyramid pooling and context-aware initialization, Gaussian-DETR improves long-event retrieval under the tested QVHighlights protocol. Experiments also show limited change in overall recall and a modest short-event trend, characterizing Gaussian-DETR as a scale-specific temporal prior module with its clearest empirical support on long-event retrieval. Looking forward, the broader principle of explicit temporal-prior injection could be explored in recent large vision-language models and end-to-end video foundation models, where Gaussian or mixture-based temporal hypotheses may provide controllable temporal structure for long-video grounding. Such extensions would require joint optimization and evaluation within foundation-model feature extraction and training pipelines and are left for future work. These results position Gaussian temporal priors as a parameter-efficient mechanism for studying structurally guided query initialization within DETR-like video moment retrieval architectures.

## Limitations and future work

Gaussian-DETR studies explicit temporal priors within a pre-extracted feature framework, where the underlying visual encoder is not jointly optimized according to feedback from the Gaussian temporal priors. This decoupled setting of feature extraction and temporal decoding may limit the model’s performance upper bound in end-to-end complex action localization tasks.

**Event-structure limitations.** Each Gaussian query encodes a unimodal temporal hypothesis through a single center and span, making the current parameterization most directly aligned with temporally continuous single-interval moments. The complete query set can cover multiple temporal regions, but fragmented or irregularly distributed evidence would require coordinated multi-query representations or richer prior families. Concurrent or strongly overlapping events similarly motivate asymmetric or mixture-based temporal priors, together with query-coordination mechanisms, to model fragmented, overlapping, and multi-instance event structures more flexibly.

**Benchmark coverage and protocol scope.** The main empirical evidence comes from QVHighlights, where Gaussian-DETR shows its most consistent benefit on Long mAP. The Charades-STA experiment provides an auxiliary same-pipeline check under the available reproduction setting. Extending the evaluation to ActivityNet Captions [28] and additional long-video benchmarks remains future work, as these datasets require separate feature-extraction and training protocols.

Future work will primarily focus on three directions: exploring adaptive distribution modeling mechanisms to enhance the flexibility of prior shapes for fragmented or asymmetrical action boundaries; extending the approach to online or streaming settings where causal feature updates and incremental Gaussian query initialization are required; and adapting explicit position-aware initialization to long-sequence architectures, such as state space models, to improve temporal modeling efficiency for complex video events.

## Supporting information

S1 Appendix Diagnostic best-per-metric analysis. This file reports the independent best-per-metric diagnostic analysis for Moment-DETR and Gaussian-DETR on the fixed five-seed subset.
(PDF)

S2 Appendix Training dynamics analysis of long-term performance. This file provides the representative-seed training-dynamics analysis for R1@0.5 and Long mAP on QVHighlights.
(PDF)
