# Supplementary material for: Explicit temporal inductive biases for video moment retrieval via multi-scale Gaussian priors
Source: PLoS One. 2026 Sep 1;21(9):e0354458. doi: 10.1371/journal.pone.0354458 (PMC13533423; doi:10.1371/journal.pone.0354458)
Supplement: Supplementary file 1 [file pone.0354458.s001.pdf]

S1 Appendix. Diagnostic best-per-metric analysis

Tables 1 and 2 report the highest value observed for each metric during training, with checkpoints selected independently for each metric on the fixed five-seed subset (42, 2022, 2023, 2024, and 2025). These results characterize metric-specific training dynamics rather than results from a single unified checkpoint. The primary performance analysis remains the 15-seed comparison based on checkpoints selected by validation R1@0.5.

Diagnostic best-per-metric analysis of Moment-DETR

By tracking performance over the complete training cycle, we observe that metric-specific peaks may differ from the checkpoint selected by the standard validation R1@0.5 criterion. Taking seed 2024 as an example, Long mAP reaches 42.64 at epoch 18 but is 39.95 at the checkpoint selected by validation R1@0.5. This example illustrates the temporal separation between metric-specific peaks and the main checkpoint-selection criterion.

Table 1 summarizes the highest observed value of the baseline model for each metric under independent best-per-metric checkpoint selection. Compared with the main-protocol results, these values show that Long mAP and Short mAP can reach higher values at some epochs, highlighting metric-specific training dynamics.

Table 1: **Diagnostic best-per-metric results of Moment-DETR on the fixed five-seed subset.**

Metric	Mean	Std	Min	Max	Notes
R1@0.5	55.28	0.44	54.84	55.87	Highest observed value during training.
Long mAP	41.29	1.07	40.03	42.64	Highest observed value during training.
Short mAP	4.37	0.33	3.96	4.84	Highest observed value during training.

Table 2: **Diagnostic best-per-metric results of Gaussian-DETR on the fixed five-seed subset.**

Metric	Mean	Std	Min	Max	Notes
R1@0.5	55.56	0.92	54.06	56.45	Highest observed value during training.
Long mAP	44.06	1.31	42.45	45.32	Highest observed value during training.
Short mAP	4.59	0.25	4.26	4.93	Highest observed value during training.

Diagnostic best-per-metric analysis of Gaussian-DETR

In comparison, Gaussian-DETR shows different convergence patterns across metrics. Overall recall and scale-specific mAP do not peak at the same stage of training. This pattern suggests that multi-scale Gaussian queries affect overall retrieval and scale-specific localization differently under the independent best-per-metric view.

In this diagnostic view, Gaussian-DETR shows higher metric-specific peak values. These values characterize training dynamics, while the main conclusions remain based on the R1@0.5-selected checkpoints.
