# Supplementary material for: Explicit temporal inductive biases for video moment retrieval via multi-scale Gaussian priors
Source: PLoS One. 2026 Sep 1;21(9):e0354458. doi: 10.1371/journal.pone.0354458 (PMC13533423; doi:10.1371/journal.pone.0354458)
Supplement: Supplementary file 2 [file pone.0354458.s002.pdf]

S2 Appendix. Training dynamics analysis of long-term performance

To further examine the convergence patterns discussed in the main text and S1 Appendix, this section visualizes and analyzes the training dynamics of the baseline model (Moment-DETR) and our method (Gaussian-DETR) on the R1@0.5 and Long mAP metrics.

As shown in Fig 1, the overall recall rate of the baseline model exhibits a saturation trend in the early stages of training, while its long-term retrieval metric (Long mAP) remains relatively flat throughout the entire training cycle, showing no significant performance growth. In contrast, Fig 1 shows that Gaussian-DETR reaches a higher Long mAP peak in the middle and late stages of training, and at this time, the overall recall rate is still in a process of dynamic evolution.

This pattern is consistent with the Gaussian temporal priors affecting the training dynamics of overall recall and long-event retrieval differently. Because Fig. 1 presents a representative seed and the two metrics peak at different epochs, this analysis complements rather than replaces the main 15-seed comparison based on R1@0.5-selected checkpoints.

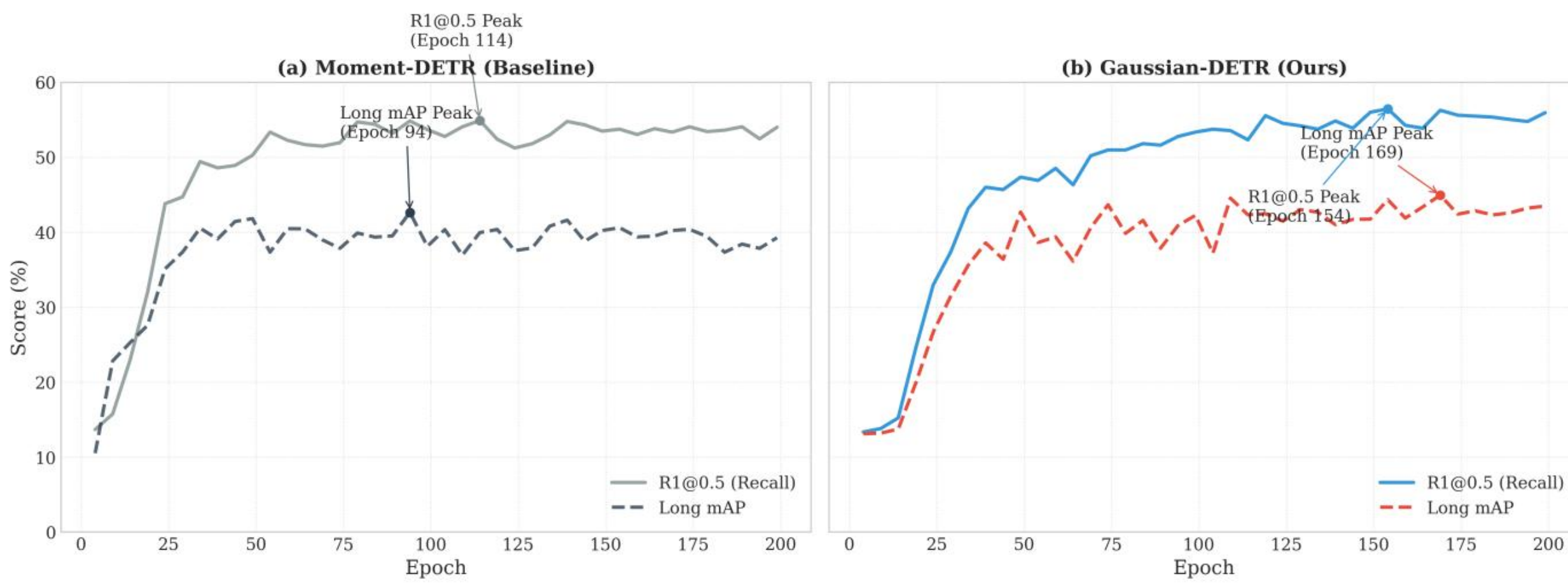

Figure 1: **Training dynamics of R1@0.5 and Long mAP on QVHighlights for a representative seed (Seed 2024).**
